# From Merkel Cell Polyomavirus Infection to Merkel Cell Carcinoma Oncogenesis

**DOI:** 10.3389/fmicb.2021.739695

**Published:** 2021-09-08

**Authors:** Nathan A. Krump, Jianxin You

**Affiliations:** Department of Microbiology, Perelman School of Medicine, University of Pennsylvania, Philadelphia, PA, United States

**Keywords:** Merkel cell polyomavirus, Merkel cell carcinoma, persistence, innate immune response, integration, dysbiosis, oncogenesis

## Abstract

Merkel cell polyomavirus (MCPyV) infection causes near-ubiquitous, asymptomatic infection in the skin, but occasionally leads to an aggressive skin cancer called Merkel cell carcinoma (MCC). Epidemiological evidence suggests that poorly controlled MCPyV infection may be a precursor to MCPyV-associated MCC. Clearer understanding of host responses that normally control MCPyV infection could inform prophylactic measures in at-risk groups. Similarly, the presence of MCPyV in most MCCs could imbue them with vulnerabilities that-if better characterized-could yield targeted intervention solutions for metastatic MCC cases. In this review, we discuss recent developments in elucidating the interplay between host cells and MCPyV within the context of viral infection and MCC oncogenesis. We also propose a model in which insufficient restriction of MCPyV infection in aging and chronically UV-damaged skin causes unbridled viral replication that licenses MCC tumorigenesis.

## Introduction

Merkel cell polyomavirus (MCPyV) infection can be detected on the skin of most healthy adults (Tolstov et al., 2009; Schowalter et al., 2010), yet details of its virology and infectious cycle remain sparse. Evidence from serological studies suggests that MCPyV infects most people during early childhood with exposure to the virus increasing as populations age (Chen et al., 2011; Viscidi et al., 2011; Martel-Jantin et al., 2013). A vast majority of MCPyV infections are asymptomatic (Tolstov et al., 2011), but some result in an aggressive neuroendocrine skin cancer called Merkel cell carcinoma (MCC) (Feng et al., 2008; Gjoerup and Chang, 2010; Harms, 2017; Schadendorf et al., 2017). The etiology of over 80% of MCC tumors can be traced to MCPyV by the presence of integrated MCPyV genomic sequence in the cellular DNA (Feng et al., 2008; Santos-Juanes et al., 2015).

Though MCC cases are rare, the incidence of MCC has tripled over the last two decades and is projected to increase further in the future (Fitzgerald et al., 2015; Paulson et al., 2018a; Stang et al., 2018; Freeman et al., 2019; Jacobs et al., 2020). MCC has a high rate of mortality with 5-year overall survival around 51% for patients presenting with local disease at the time of diagnosis, and worse prognoses for those with more advanced stages of disease (Harms K. L. et al., 2016). Primary MCC malignancies are combatted by surgical resection, sentinel lymph node biopsy, and/or adjuvant radiation (Cassler et al., 2016). In metastatic MCC, chemotherapies have thus far failed to produce durable responses (Iyer et al., 2016; Cowey et al., 2017). Promisingly, a recent bourgeoning of anti-PD1 and anti-PDL1 treatments for MCC has yielded prolonged responses in patients with advanced disease, though a significant proportion of patients do not respond and the durability of responses varies (Iyer et al., 2016; Becker et al., 2017; D'angelo et al., 2018; Nghiem et al., 2019). The pursuit of more targeted and effective MCC therapies necessitates a better understanding of the oncogenic underpinnings of MCC and the role of MCPyV in tumorigenesis.

Despite the status of MCPyV as a member of the human skin virome and its relevance to human disease, much is unknown about its biology. For instance, while it is clear that MCPyV integration and oncogene expression enable MCC cell growth, the conditions that lead to MCPyV integration are not known. Neither has a consensus been reached regarding the MCC cell of origin. Most pressingly, methods of limiting MCPyV infection and thereby preventing MCC onset have yet to be discovered. In recent years, however, efforts have been aimed at exploiting the presence of MCPyV oncoproteins in MCCs to develop targeted therapies for virus-positive MCC tumors (Chapuis et al., 2014; Gavvovidis et al., 2018; Longino et al., 2019; Sarma et al., 2020). The potential for expanding on these opportunities to provide prophylactic or therapeutic interventions for a highly lethal skin cancer should not be overlooked.

### Merkel Cell Carcinoma

MCC was first described in 1972 by Cyril Toker, MD (Toker, 1972). He and his colleagues discovered that the tumors arose in the dermis or lower subcutis layers of the skin and that they metastasized readily via the lymphatic system. Because the malignancies shared neuroendocrine markers with Merkel cells, such as cytokeratin-20 (CK20) and neuron-specific enolase (NSE), the disease was named Merkel cell carcinoma (De Wolff-Peeters et al., 1980; Gu et al., 1983; Moll et al., 1992).

The next breakthrough in characterizing MCC came in 2008 when the Chang and Moore group identified a novel polyomavirus genome monoclonally integrated in the DNA of MCC tumor cells (Feng et al., 2008). Previously, they had correctly predicted the viral etiology of AIDS-associated Kaposi's sarcoma (KS) (Chang et al., 1994), hypothesizing that the altered immune status of these individuals engendered dysbiosis between the host and virus to trigger oncogenesis. Following the same principle, they suspected viral involvement in MCCs given the knowledge that MCCs were far more likely to occur in HIV-positive individuals (Engels et al., 2002). Probing MCC primary tumors and metastases by digital transcriptome subtraction and viral genome walking revealed the presence and sequence of the MCPyV genome, respectively (Feng et al., 2008).

As a result of their discovery, assays to determine the status of MCPyV have provided definitive markers and more assured diagnosis for a subset of MCCs (Buck and Lowy, 2009; Decaprio, 2009; Duncavage et al., 2009; Haugg et al., 2014; Starrett et al., 2020). Moreover, it is now widely acknowledged that immunocompromised individuals are more likely to develop virus-driven cancers (Ellerbrock et al., 2000; Weber et al., 2006; Chadburn et al., 2013; Ponce et al., 2014; Schadendorf et al., 2017). The discovery of MCPyV also opened the opportunity to research the role of MCPyV oncogenes in MCC carcinogenesis and to inform our understanding of human cancer.

Building upon those earlier discoveries, recent research has been aimed at comparing MCPyV-positive and MCPyV-negative MCCs, which despite evidently different etiologies, have similar disease presentation and prognoses (Fischer et al., 2010; Handschel et al., 2010; Schrama et al., 2011). In virus-associated MCC, MCPyV DNA is integrated into the tumor cell genome in a manner that preserves expression of MCPyV genes called tumor (T) antigens (Shuda et al., 2008, 2011; Cheng et al., 2013). Expression of MCPyV T antigens drives oncogenesis in virus-positive MCC tumors and is required for the growth of the tumor cells (Houben et al., 2010, 2012; Shuda et al., 2011, 2014; Spurgeon and Lambert, 2013; Verhaegen et al., 2014; Grundhoff and Fischer, 2015; Wendzicki et al., 2015). MCPyV T antigens can support MCC growth and survival despite otherwise low chromosomal mutation burdens in MCPyV-positive MCCs (Starrett et al., 2017). By contrast, virus-negative MCCs exhibit a high, UV-related, mutation frequency indicating that pro-oncogenic mutations arose as a direct result of chronic exposure to UV-radiation (Harms et al., 2015; Wong et al., 2015; Goh et al., 2016; Starrett et al., 2017). MCPyV-negative MCCs also have higher levels of activation-induced cytidine deaminase (AID) which could contribute to mutagenesis (Matsushita et al., 2017). The gradual selection for transforming mutations may lead to shared traits among MCPyV-negative cancers such as loss-of-function mutations in Rb, NOTCH, PRUNE2, as well as, activating mutations in PI3KCA and HRAS (Sihto et al., 2011; Nardi et al., 2012; Harms et al., 2013, 2015; Cimino et al., 2014; Sahi et al., 2014; Wong et al., 2015; Goh et al., 2016; Harms P. W. et al., 2016).

Disparities between the etiologies of MCPyV-positive and -negative MCCs extend to differences in their morphology. MCPyV-containing malignances are more likely to have regularly-shaped nuclei, low cytoplasm volume, and more homogeneous cell types than those lacking MCPyV (Kuwamoto et al., 2011; Iwasaki et al., 2013). They are also more likely to display classical markers for MCC such as CK20 and neurofilament (Pasternak et al., 2018). Differences in morphological phenotype between the two MCC types could be a reflection of their alternative expression profiles in factors such as cell adhesion molecules or miRNAs (Xie et al., 2014; Iwasaki et al., 2016).

There are also disparities between these two types of cancers that have more immediately discernible implications. Virus-associated MCCs are more likely to occur in non-sun-exposed areas than MCPyV-negative MCC (Dabner et al., 2014; Leroux-Kozal et al., 2015) and on a population scale, virus-negative MCCs occur in greater proportions in Australia, where a significant population of individuals with low-melanin concentrations are exposed to high levels of UV-radiation (Paik et al., 2011). Moreover, MCC tumors in younger patients and females are more likely to be virus-positive (Wang et al., 2017). These trends suggest that while UV-exposure and advanced age increase the incidence of both cancer types, MCPyV oncogenesis is less dependent on these exogenous factors.

Beyond differences in oncogenic mechanism, morphology, and incidence, there are an increasing number of reports that MCPyV-positive MCC patient prognosis is statistically better than those with MCPyV-negative MCC (Sihto et al., 2009; Laude et al., 2010; Higaki-Mori et al., 2012; Leroux-Kozal et al., 2015; Moshiri et al., 2017). One reason for the improved patient survival associated with MCPyV-LT-expressing MCC tumors may be correlated with the presence of foreign T antigens that enhance immunogenicity (Walsh et al., 2016). In MCC patients undergoing PD-1 treatment, there is a greater degree of B and T cell clonality in MCPyV-positive tumor infiltrates than MCPyV-negative, reflecting the greater diversity of neoantigens in the latter case (Miller et al., 2018). MCPyV-positive MCCs also exhibit elevated expression of prokineticin-2, an inflammatory and angiogenic signaling molecule, resulting in enhanced T cell infiltration (Lauttia et al., 2014). Another reason for better prognoses in MCPyV-positive MCC cases could be that lower frequency of somatic mutations equate to expression of wild type tumor suppressors like p53 that support therapeutic interventions and native immune responses in restricting cancer progression.

The juxtaposition of these two MCC subtypes informs our understanding of both through comparative analysis, and can guide the development of novel treatments. In MCPyV-driven MCC, there exists the promise of truly targeted therapies toward an aggressive solid tumor. What we learn about viral MCCs can be applied to the more complicated and divergent cases of MCPyV-negative MCC. These lessons can, in turn, broaden our understanding of this family of rapidly metastasizing skin cancer.

### MCPyV Genome and Encoded Genes

#### MCPyV Genome

**Figure 1:**
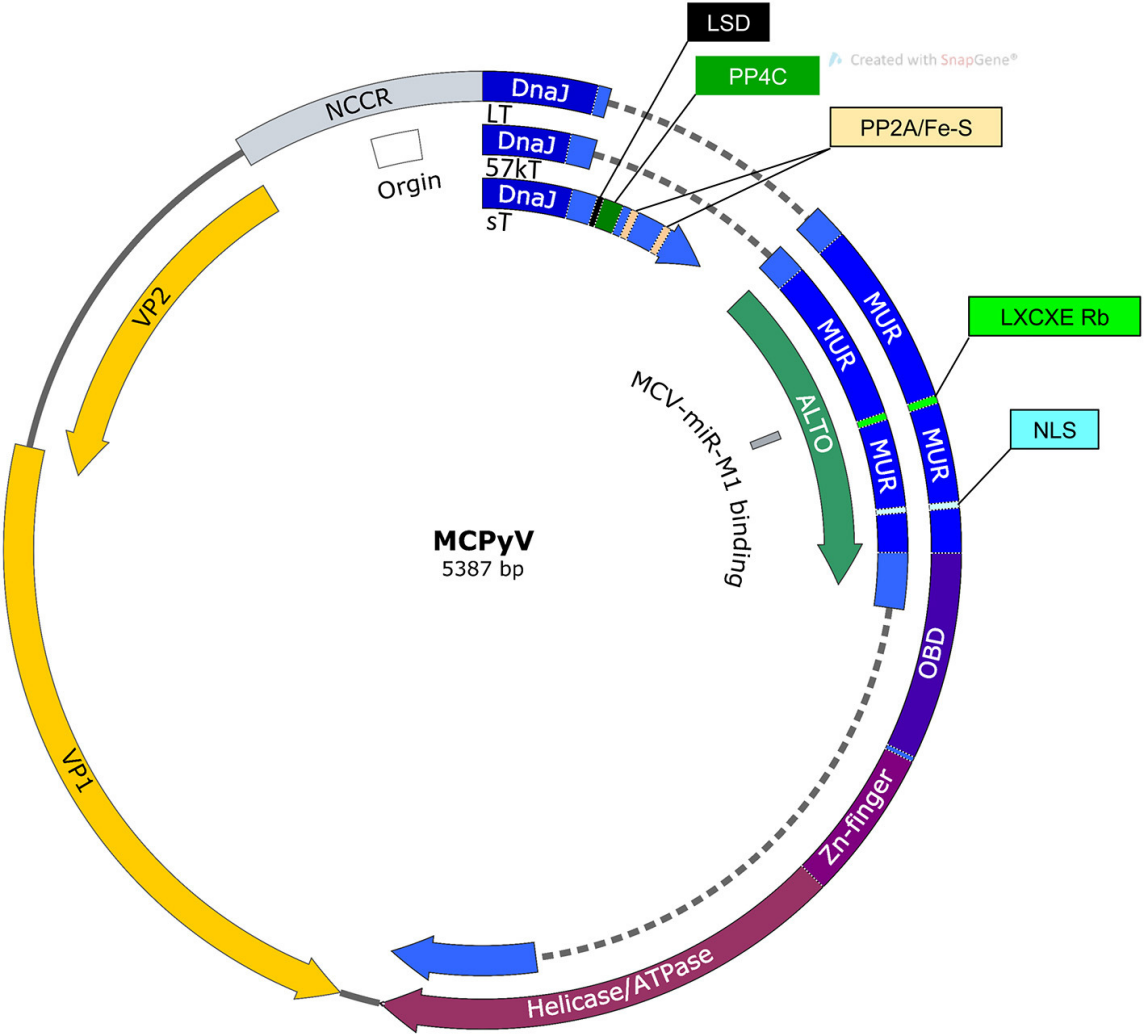
Map of MCPyV genome. NCCR, Non-coding control region; Origin, Origin of replication; LSD, LT stabilization domain; NLS, Nuclear localization signal; MUR, MCPyV unique region; OBD, Origin binding domain.

MCPyV has a ~5.4 kb circular dsDNA genome (Figure 1). Like other polyomaviruses, existing evidence suggests that the MCPyV genome remains episomal throughout the infectious cycle (Gjoerup and Chang, 2010; Liu et al., 2016a,b). The viral genome is divided into early and late regions by a non-coding control region (NCCR) containing the viral origin of replication and bidirectional promoters that drive early and late gene transcription (Harrison et al., 2011). The early region expresses alternatively spliced tumor antigens, termed large tumor antigen (LT) and small tumor antigen (sT) that support replication, as well as, 57kT and an alternate LT open reading frame (ALTO) with functions that are less defined (Kwun et al., 2009; Carter et al., 2013). Major and minor capsid proteins, VP1 and VP2, respectively, are expressed from the MCPyV late region along with a miRNA that has been proposed to modulate early gene expression (Seo et al., 2009; Schowalter et al., 2011).

In MCPyV-positive MCC, the MCPyV genome is integrated into the host DNA such that the functions of its early promoter and partial expression of its T antigens are preserved (Feng et al., 2008). Point mutations in other regions of the genome and truncations of the MCPyV LT C-terminal domain, however, are common in viral MCCs (Liu et al., 2016a). Expression of the viral oncoproteins is largely driven by the preserved MCPyV promoter rather than endogenous promoters, though there are conflicting reports as to whether MCPyV is more likely to integrate into specific regions of chromatin (Doolittle-Hall et al., 2015; Czech-Sioli et al., 2020). Sequencing of integration sites in multiple MCC tumors reveal that initial recombination of a linearized MCPyV genome with the host genome could lead to transient circularization and amplification of the viral genome and neighboring host DNA (Starrett et al., 2017). The amount of amplification and the site of DNA repair accounts for differences in viral genome copy number and duplications of host sequences.

#### Large Tumor Antigen

In MCPyV infected cells, LT localizes to the nucleus where it performs functions directly and indirectly supporting MCPyV replication (Nakamura et al., 2010). Like T antigens in other polyomaviruses, LT contains an origin binding domain (OBD) and an ATP-dependent helicase domain by which it unwinds MCPyV DNA for replication (Harrison et al., 2011). LT localizes to replication foci containing high concentrations of nascently synthesized MCPyV genomes (Liu et al., 2016b). In replication foci, LT binds to G(A/G)GGC-like pentanucleotide sequences on the MCPyV genome to initiate efficient replication in a manner that requires the LT DnaJ domain and is supported by the presence of sT (Kwun et al., 2009; Harrison et al., 2011).

Somatic genes also localize to MCPyV replication foci and support LT-mediated replication. Bromodomain protein-4 (BRD4) associates with LT in replication centers where it amplifies MCPyV replication by recruiting replication factor C (RFC) (Wang et al., 2012). Ataxia telangiectasia mutated (ATM) and Rad3-related (ATR) DNA damage response (DDR) factors also co-localize to MCPyV replication centers in a manner dependent on the presence of LT and the MCPyV origin (Tsang et al., 2014). These DDR proteins support efficient viral genomic DNA synthesis, but may also be essential in limiting the transforming potential of MCPyV T antigens. For example, ATM phosphorylates LT in the C-terminal domain at Ser-816, leading to increased apoptosis (Li et al., 2015). Alanine mutagenesis at this site leads to enhanced colony formation in C33A cells. Therefore, this LT-ATM interaction could both promote MCPyV replication and limit rampant cellular growth. The delicate balance of establishing an S-phase-like environment for the production of new virions without causing the terminal fate of cellular transformation can explain the duality of MCPyV traits.

The necessity of MCPyV to avoid terminating its infectious cycle by transforming its host cell is underscored by the fact that unlike other polyomavirus T antigens, LT neither binds nor inhibits p53 (Lilyestrom et al., 2006; Cheng et al., 2013). In fact, the studies by Li and colleagues showed that the helicase activity in full-length LT, in the context of MCPyV DNA synthesis, induces cell cycle arrest in a p53-dependent manner that limits cellular proliferation (Li et al., 2013). This observation provided an evolutionary explanation as to why MCPyV may have lost the ability to inactivate p53, in that allowing p53 to guard cell cycle progression could limit incidental progression to cancer and abortive MCPyV infection. It also suggests why mature MCCs invariably express a truncated variant of LT (LTT) lacking the C-terminal helicase domain. In support of the protective nature of the LT C-terminal domain, expression of LTT, but not full-length LT, sensitizes cells to UV-DNA damage due to impaired cell cycle arrest and DDR (Demetriou et al., 2012). Moreover, expression of LTT promotes cell growth, while expression of the C-terminal domain alone, or full-length LT, negatively regulates cell growth (Cheng et al., 2013).

The ability of MCPyV LTT to promote cellular proliferation has been attributed to its ability to bind and inactivate Rb through an LXCXE domain similar to other polyomaviruses (Houben et al., 2012). This region is present in both wild-type LT and LTT. The LT-Rb interaction results in enhanced E2F-transcriptional activity; promoting growth in MCPyV-positive MCC cells (Sihto et al., 2011; Hesbacher et al., 2016; Schrama et al., 2016). The impact of LT-Rb binding was further illustrated by the fact that silencing LTT expression in a xenograft MCC mouse model resulted in tumor regression in an Rb-binding dependent manner (Houben et al., 2012). The LXCXE-Rb interaction also enhances entry into S-phase, cellular proliferation, and motility in hTERT immortalized BJ human foreskin fibroblasts (BJ-hTERT) (Richards et al., 2015). The functional significance of MCPyV LTT inactivation of Rb in human populations is also supported by the finding that MCPyV-negative MCCs frequently contain mutations in the Rb gene, whereas MCPyV-positive tumors usually express wild type Rb (Sihto et al., 2011).

LT-Rb binding domain could also be responsible for increasing Sox2 and subsequent Atoh expression in MCPyV-positive MCC cells (Harold et al., 2019). This is significant because the activity of these transcription factors can confer cells with markers of the shared phenotype between Merkel cells and MCCs both *in vitro* and *in vivo* (Verhaegen et al., 2017). Though sT has garnered more attention regarding cellular transformation, it may be that LT alters the transcriptional landscape of the original cell of MCC to imbue it with its morphological characteristics.

Recently, it has been shown that the Rb inhibitory domain of LT could indirectly activate p53 by upregulating ARF, an inhibitor of the p53-degrading E3 ubiquitin ligase MDM2 (Park et al., 2019). This assertion suggests that even without the helicase domain, LTT would activate p53 in MCC cells, but the investigators propose that the sT-MYC-EP400 transcriptional complex counteracts p53 activity through upregulation of MDM2 and the related enzyme MDM4 (Park et al., 2019).

#### Small Tumor Antigen

MCPyV sT consists of 186 amino acids, including a C-terminus that is spliced out of the other MCPyV T antigens that confers it with entirely unique functions. MCPyV sT localizes to the nucleus and is able to support LT-mediated replication though the exact mechanism remains unclear (Kwun et al., 2009). The functions carried out by the C-terminal domain unique to MCPyV sT is of critical importance because its expression is necessary for MCC survival and appears to be the primary driver of cellular transformation (Shuda et al., 2011).

MCPyV sT expression is capable of transforming rat fibroblasts and epithelial cells in an *in vivo* mouse model via a region incorporating amino acids 91–95 termed the LT stabilization domain (LSD) (Shuda et al., 2011; Kwun et al., 2015; Verhaegen et al., 2015). As its name implies, MCPyV sT containing wild-type LSD increases LT protein level, though the underlying mechanism is an area of active investigation (Kwun et al., 2013; Dye et al., 2019). MCPyV sT drives cellular transformation in rat fibroblasts by promoting hyperphosphorylation of eukaryotic translation-initiation factor 4E-binding protein (4E-BP1) (Shuda et al., 2011; Wu et al., 2015; Velasquez et al., 2016). The LSD domain was also linked to activation of the non-canonical NF-κB pathway, induction of a senescence associated secretory phenotype (SASP), and enhanced MCC cell proliferation (Zhao et al., 2020). Besides those functions attributed to the LSD domain, numerous other oncogenic functions have been ascribed to MCPyV sT.

MCPyV sT expression may promote cellular growth by activating c-Jun downstream of MEK/ERK factors (Wu et al., 2016). In addition, MCPyV sT expression in normal fibroblasts elevates aerobic glycolysis via modulation of the host cell transcriptome, including upregulation of monocarboxylate lactate transporter SLC16A1 (MCT1), likely contributing to oncogenic potential (Berrios et al., 2016). Still another way in which sT could enhance metastatic potential is through disruption of inter-cellular junctions via upregulation of A-disintegrase-and-metalloproteinase (ADAM) 10 and 17, which are more highly expressed in MCPyV-positive MCCs (Nwogu et al., 2018). The manner in which MCPyV sT might be affecting transcriptional changes described above is by recruiting a MYCL-MAX heterodimer to the EP400 complex (Cheng et al., 2017). This interaction was elegantly shown to promote cell viability in MCPyV-positive cell lines, MKL-1 and WaGa, as well as, confer a transforming phenotype in keratinocytes (Cheng et al., 2017). Two genes that are upregulated by this transcriptional program are MDM2, which promotes p53 proteasomal degradation, and lysine-specific histone demethylase 1A (LSD1), which is necessary for maintaining plasticity and proliferative capacity of MCC cells (Park et al., 2019, 2020; Leiendecker et al., 2020). Importantly, inhibition of MDM2 and LSD1 induces cell death in MCC cells and reduces the growth of MCC tumor in mice, demonstrating the therapeutic potential of using MDM2 and LSD1 inhibitors in treating this highly aggressive skin cancer (Park et al., 2019, 2020; Leiendecker et al., 2020).

MCPyV sT contains protein phosphatase 2A (PP2A) binding sites similar to other polyomaviruses, but this binding activity is not required for transformation of rat fibroblasts (Kwun et al., 2015). It has been suggested that these PP2A binding sites are involved in the interaction with protein phosphatase 4C (PP4C) (Griffiths et al., 2013; Kwun et al., 2015). In addition to findings mentioned earlier, one group found that sT targets NEMO through interaction with its regulatory subunit (PP4R1) to disrupt NF-κB mediated inflammatory signaling (Griffiths et al., 2013; Abdul-Sada et al., 2017). The sT-PP4C interaction has also been implicated in lowering microtubule stability through altered expression of cellular proteins like stathmin, as well as, Rho GTPase-mediated actin remodeling, leading to an enhanced cell motility phenotype (Knight et al., 2015; Stakaityte et al., 2018). These broad changes resulting from the sT-PP4C interaction have been recently ascribed to upregulated p38 MAPK signaling via MKK4 (Dobson et al., 2020). MCPyV sT related motility might therefore confer transformed cells with invasive and metastatic properties.

Our group found that the proposed PP2A binding sites might also serve as iron-sulfur (Fe/S) cluster domains (Tsang et al., 2015). This discovery had significance because proteins containing Fe/S domains often modulate helicase activity (Pugh et al., 2008; Wu and Brosh, 2012). The function of MCPyV sT Fe/S clusters was linked with its localization to LT-containing replication foci and enhanced MCPyV DNA synthesis without increasing LT protein stability. Moreover, MCPyV sT was able to coordinate Fe/S much more efficiently than sT proteins from SV40, HPyV6, and HPyV7. Another group recently found that expression of MCPyV sT in HEK 293 cells elevated several markers of DNA damage, and at a higher rate than HPyV6 and HPyV7 sT proteins (Wu et al., 2019). Given these findings, the supportive role of sT in LT-mediated MCPyV replication could be direct, through sT activity in replication centers, or indirect, through activation of ATM and subsequent phosphorylation of LT Ser816.

#### 57kT and ALTO

A third alternatively spliced T antigen is that of 57kT (Figure 1). 57kT does not appear to support MCPyV replication, and its specific function in infection or in MCC oncogenesis is unclear (Kwun et al., 2009). Because it shares the MCPyV unique region (MUR) with LT, but lacks the helicase and origin-binding domains, it is reasonable to hypothesize that 57kT indirectly supports MCPyV infection, but does not directly support replication as LT does. Similar to LTT, 57kT could bolster pro-oncogenic functions associated with Rb-binding without helicase-related cell cycle arrest or DNA damage.

Carter and colleagues discovered an overprinting gene product expressed from the early region that they termed alternate Large T open reading frame (ALTO) (Carter et al., 2013) (Figure 1). This transcript utilizes a start codon that is +1 nucleotide frame-shifted relative to the second exon of LT. The authors hypothesize that overprinting genes such as these evolve in viruses as a strategy to maximize the coding potential of relatively small genomes (Carter et al., 2013). Recently, several ALTO-encoding circular RNAs have been identified in MCPyV-positive MCC cell lines and tumor tissues (Yang et al., 2021). MCPyV ALTO translated from these circular RNAs has the ability to transactivate recombinant promoters, as well as, a number of key cellular genes involved in MCPyV pathogenesis. Therefore, MCPyV ALTO protein may be able to modulate MCPyV infectious and tumorigenic potential through transcription regulation (Yang et al., 2021).

#### VP1, VP2, and miR-M1

The MCPyV late promoter drives expression of the capsid proteins VP1 and VP2, as well as, a miRNA, MCPyV miR-M1 (Figure 1). VP1 and VP2 encapsidate MCPyV DNA during packaging and mediate cell surface interactions that activate entry and trafficking (Schowalter et al., 2011; Neu et al., 2012; Schowalter and Buck, 2013). Attachment to cell surfaces is mediated through interactions between VP1 capsomeres and sulphated polysaccharides while subsequent viral entry requires the interaction between VP1 and sialic acid (Neu et al., 2012; Bayer et al., 2020). In A549 cells, MCPyV entry was mediated through caveolar/lipid raft endocytosis (Becker et al., 2019). Subsequent MCPyV trafficking is carried out by the endosomal-to-ER pathway requiring microtubule transport activity (Becker et al., 2019). By comparison, VP2 mediates post-attachment phases of MCPyV entry and is necessary for native MCPyV infection, if not the formation of pseudo-virus particles (Schowalter and Buck, 2013).

Another late region gene product MCPyV miR-M1 likely regulates MCPyV early gene expression. In MCPyV-transfected neuroectodermal tumor (PFSK-1) cells, highly expressed miR-M1 transcripts down-regulated the expression of MCPyV T antigens and MCPyV replication (Theiss et al., 2015). The function of miRNA-M1 to restrain MCPyV replication and gene expression enabled a low level of detectable MCPyV to persist in cells for several months. Thus, the MCPyV miRNA could drive a persistence mechanism by which the virus maintains a limited infection in human hosts for prolonged periods. In addition, it was found that expression of a synthetic MCPyV-miR-M1 in HEK293 or MCC cells targets the cellular host gene SP100 thereby lowering CXCL8 expression and neutrophil chemotaxis (Akhbari et al., 2018). In principle, this anti-inflammatory effect could be protective in the setting of MCPyV infection. Unlike analogous miRNAs in animal polyomavirus tumors, however, the MCPyV miRNA is not highly expressed in MCC tumors, suggesting that the function for which it is selected in MCPyV infection does not, in turn, promote tumor fitness (Chen et al., 2015).

## MCPyV Tropism and a Model of Infection

Epidemiological evidence suggests that MCPyV establishes asymptomatic, persistent infections in most people. As many as 88% of healthy adults are positive for MCPyV-specific antibodies (Kean et al., 2009; Pastrana et al., 2009; Tolstov et al., 2009; Touze et al., 2011). Serological activity against the MCPyV major capsid protein increases as populations age, from about 10% in early childhood to about 80% in adults (Chen et al., 2011; Tolstov et al., 2011; Viscidi et al., 2011). MCPyV-specific antibody titers positively correlate with viral load as measured by MCPyV DNA encapsidated in viral particles shed from healthy skin, suggesting that MCPyV positivity rates could also increase as populations age (Schowalter et al., 2010; Pastrana et al., 2012).

Within a given healthy individual, MCPyV antibody titers remain relatively stable over a period of at least 15 months (Pastrana et al., 2012). By comparison, neutralizing antibody titers to MCPyV, but not other human polyomaviruses, are significantly higher in patients with MCPyV-positive MCC despite the fact that MCC tumors do not express capsid protein (Pastrana et al., 2009). Together, these findings suggest that MCPyV has the capacity to persist, and that MCPyV expansion within a host correlates with disease propensity. Inadequate restriction of MCPyV may be a critical factor in enabling MCC development. This hypothesis is supported by the fact that chronic UV-exposure, advanced age, and HIV-related or iatrogenic immunosuppression pose significant risk for MCC (Heath et al., 2008; Bertrand et al., 2013; Ma and Brewer, 2014).

Direct evidence of the natural host reservoir cells that maintain latent MCPyV infection remains elusive. To take steps in establishing the cellular context of MCPyV infection, we discovered that primary human skin dermal fibroblasts support productive MCPyV infection *in vitro* and *ex vivo* (Liu et al., 2016b). While developing the *in vitro* model, we found that the addition of modulatory factors present in the skin could greatly enhance MCPyV proliferation. Specifically, viral entry is stimulated during the first 2 days of infection in the absence of serum and in the presence of collagenase. This is analogous to the inflammatory skin in which matrix metalloproteinases (MMPs) digest collagen fibers and activate chemokines (Werner and Grose, 2003; Gill and Parks, 2008). Subsequently, MCPyV replication is boosted by priming human dermal fibroblasts with epidermal and fibroblast growth factors and a WNT activator, which induce fibroblast proliferation and MMP expression (Liu et al., 2016b). That these same conditions are found in the skin wounding response suggests that the MCPyV infection cycle could be linked to damage to the skin by way of abrasions or UV-damage.

The picture of MCPyV infection is far from complete, yet inferences can be made from distinct sources of evidence. In *ex vivo* skin culture, MCPyV preferentially infects dermal fibroblasts underlying the basal layer of the epidermis and those surrounding hair follicles (Liu et al., 2016b). Furthermore, MCPyV virions are readily detected in eyebrow hair bulbs sampled from healthy human volunteers (Peretti et al., 2014; Bellaud et al., 2015; Hampras et al., 2015). It is possible that MCPyV infects the dermal cells surrounding the hair follicle and subsequently uses the follicular space as a means to disseminate to the skin surface and access new hosts. In addition, migration of MCPyV-infected fibroblasts to wound sites could represent another mode of transmission from reservoir cells into the deeper layers of the skin.

MCPyV may also infect cells at body sites other than the skin to establish a reservoir (Salakova et al., 2016). For instance, MCPyV DNA was detected in buffy coats of healthy blood donors and inflammatory monocytes of MCC patients, indicating that the virus may establish latent infection in peripheral blood leukocytes (Mertz et al., 2010; Pancaldi et al., 2011). In two MCPyV-positive patients with prior history of MCC and active non-melanoma/non-MCC skin cancers respectively, MCPyV DNA was detected in inflammatory, but not resident monocytes (Mertz et al., 2010). Presence of MCPyV in inflammatory monocytes of patients with distinct medical histories suggests that the virus may reside in these cells and use them to spread throughout the body (Mertz et al., 2010).

There is no animal model for MCPyV infection; a goal which has proven to be challenging due to the narrow host range of the virus (Liu et al., 2018). Lack of an animal model for MCPyV infection presents a major obstacle for identifying potential reservoirs and elucidating the MCPyV infectious cycle. Generation of MCPyV chimeras with mammalian polyomaviruses may provide a solution to overcome its narrow host range in the future (Liu et al., 2018).

## MCC Cell of Origin

Though named for similarities to Merkel cells, the true original cell of MCC is rigorously debated. The proposition that MCC arises from differentiated Merkel cells is in question because these cells are post-mitotic, and thereby have limited oncogenic potential, and because they arise in the epidermis, while MCCs almost always occur in the dermis or subcutis layers (Toker, 1972). In response, it has been suggested that MCCs could arise from Merkel cell progenitor cells present at the hair follicle (Zur Hausen et al., 2013; Sauer et al., 2017; Narisawa et al., 2019). Similarly, progenitor cells derived from the neural crest have been pointed to since MCPyV-positive MCC cell lines cocultured with keratinocytes undergo neuronal morphological differentiation in a manner dependent on MCPyV LT upregulation of Sox2 and Atoh1 (Harold et al., 2019).

Some also propose that MCC could have an epithelial origin since on rare occasions, epithelial MCCs have been reported (Narisawa et al., 2020; Navarrete-Dechent et al., 2020; Song et al., 2020). One group advocating for an epithelial origin of MCC points to a case of a combined MCC and trichoblastoma tumor that shared somatic mutations (Kervarrec et al., 2019). They propose that this mixed tumor could represent a transition from early to late MCC carcinogenesis in cells with integrated MCPyV genome beginning to predominate. The same group also found that expressing MCPyV sT and GLI1 in keratinocytes results in an MCC-like phenotype, including expression of CK20 (Kervarrec et al., 2020). Another group has developed a mouse model for MCC, by expressing MCPyV sT and Atoh in keratinocytes, that results in the epidermal layer developing several MCC markers and characteristics (Verhaegen et al., 2017).

It has also been argued that pre/pro B cells are the source of MCC because MCC cells consistently express a number of B-lymphoid lineage markers, like Pax5 and TdT (Zur Hausen et al., 2013; Sauer et al., 2017). Cell expression similarities may be coincidental however, since it has also been proposed that epigenetic changes in the cell of origin could lead to a dramatic transcriptional and phenotypic changes culminating in Merkel cell resemblance. It has even been proposed that MCPyV-positive and -negative carcinomas have distinct cells of origin, and that through epigenetic reprogramming they converge on a common phenotype (Sunshine et al., 2018). Under normal developmental conditions, loss of polycomb repressive complex 2 (PRC2) and subsequent reduction H3K27me3 marks enables the differentiation of Merkel cells in mice (Bardot et al., 2013; Perdigoto et al., 2016). Given this information, one group reasoned that the development of MCC may involve a similar change in the epigenome of the unknown cell of origin. They found that pure MCPyV-positive MCCs were more likely to have lower H3K27me3 than MCPyV-negative tumors (Busam et al., 2017). Other researchers, however, found contradicting evidence that virus-negative MCCs, especially those with combined squamous cell carcinomas, had lower H3K27me3 marks than MCPyV-positive MCCs (Matsushita et al., 2019).

## Immune Responses to MCC

Immune function is relevant to every aspect of MCC progression. Incidence of MCC is greatly increased in immunocompromised individuals, especially in those who are HIV-positive or recipients of organ transplants (Koljonen et al., 2009; Ma and Brewer, 2014; Cook et al., 2019). Loss of adaptive immune competence increases the risk of both MCPyV-positive and negative tumors, implying that immune surveillance of nascently transformed MCC cells is protective at early stages of the disease. There is also evidence that adequate immune responses can lessen the severity of MCC disease progression. For example, patients with chronic inflammatory disease had higher rates of MCC incidence, and the tumors of those that developed MCC had higher expression of the proliferative marker Ki-67, and a greater tumor size (Sahi et al., 2017). Furthermore, systemic immune suppression has been linked to greater incidence of MCC, as well as, lower rates of survival in MCC patients (Paulson et al., 2013). These epidemiological data suggest that immune restriction of MCC is critical to patient outcomes at each stage of the disease.

Studies of the immunological interface in MCC tumors have revealed further detail regarding the course of disease and the ability of MCC to evade destruction. For example, circulating MCPyV T antigen-specific antibody level positively correlates with MCPyV-positive MCC recurrence and has prognostic value (Samimi et al., 2016; Paulson et al., 2017). In MCC tumors, CD8 T cell and other immune cell infiltration is usually poor, and those cases with better infiltration positively correlate with patient outcomes (Sihto et al., 2012; Lipson et al., 2013; Wheat et al., 2014; Feldmeyer et al., 2016; Miller et al., 2017). Moreover, the MCPyV-specific T cells present in MCC tumors expressed markers of exhaustion like PD-1 and Tim-3 (Afanasiev et al., 2013). The same phenomenon could be found *in vitro* and in a MCC xenograft mouse-model (Dowlatshahi et al., 2013). Histopathology of MCC tumors indicate that immune cells expressing exhaustion markers like PD-1 and CD33 congregate in the areas surrounding the tumors, suggesting that MCC acquires traits that suppress immune cell migration (Mitteldorf et al., 2017).

Observations that tumor-experienced T cells were unable to infiltrate MCC tumors led to recent efforts to introduce immune checkpoint therapies in metastatic cases. A 2015 case study showed a promising response in a patient with metastatic MCC case treated with anti-PD1 antibodies (Mantripragada and Birnbaum, 2015). In the years that followed, clinical trials exploring safety and efficacy of anti-PD1 and anti-PDL-1 infusions yielded significant response rates and relatively low rates of adverse events given the severity of patient prognosis (Iyer et al., 2016; Kaufman et al., 2016; Nghiem et al., 2019). While the durability of responses varied, they could last well over a year (Kaufman et al., 2018; Nghiem et al., 2019).

Given that there are no effective chemotherapeutic treatments available for metastatic MCC, the introduction of immune checkpoint inhibitors greatly benefitted patients (Colunga et al., 2017; Paulson et al., 2018a). Still, a significant portion of patients do not respond or have responses that are short-lived. Moreover, patients with immunosuppression or autoimmune disease may not be eligible for immune-based therapy. It also became clear that loss of durability or relapse is a problem in long term treatment for MCC, even in MCPyV-positive MCC cases where mutagenesis is low. A longitudinal study revealed that HLA-I components were downregulated in resistant tumor cells at the level of transcription as a result of continued interaction with CD8 T cells (Paulson et al., 2018b). These shortcomings necessitate the development of alternative strategies or combination of existing therapies to modulate immunity to MCC.

One such strategy is to augment innate immunity at the primary tumor site in order to enhance adaptive function systemically. One group was able to achieve improved responses at distal MCC metastases by injecting a TLR-4 agonist intratumorally at the primary MCC site followed by standard-of-care surgery and irradiation (Bhatia et al., 2019). They later delivered IL-12 plasmids to MCC tumors via electroporation that led to enhanced immunogenicity at primary and distal tumors in all patients tested; 25% of which had responses in the progression of their disease (Bhatia et al., 2020). Still other means of activating intralesional innate inflammation have proven effective. Administration of an oncolytic herpesvirus that stimulates granulocyte-macrophage colony-stimulating factor has led to durable complete responses in multiple patients (Nguyen et al., 2019; Westbrook et al., 2019). A recent case study involving an advanced-stage MCC patient resulted in complete remission upon receiving a combination of radiation therapy and anti-PD1 therapy (Bloom et al., 2019). This suggests that targeted DNA damage at accessible lesions combined with activation of immunity against a primary tumor could help train immune responses against distant lesions.

While probing the cause of immune suppression characterizing the MCC tumor microenvironment, our group discovered that STING (stimulator of interferon genes) is dramatically repressed in MCC cell lines and tumor cells (Liu et al., 2020). Among these immortalized and primary MCC cells, the marked reduction in STING expression was unique to those that were MCPyV-positive (Liu et al., 2020). Since SV40 LT has been shown to antagonize the STING signaling pathway (Lau et al., 2015), it would be valuable to determine if MCPyV LTT expressed in virus-positive MCC cells downregulates STING expression. We also showed that rescuing STING expression and activation in MCC cells led to greatly induced cytokine expression, T cell migration, and MCC cell death (Liu et al., 2020). Delivery of a mutant STING vector via AAV transduction and subsequent activation with a selective agonist could achieve the same results. Such a strategy has the potential to be highly specific in humans without the danger of systemic inflammatory pathology because the mutant-specific STING agonist does not interact with native human STING (Liu et al., 2020).

Targeting distal MCC metastases is also the aim of researchers activating or genetically engineering immune cells for autologous infusion. For example, one group showed that activation and administration of genetically engineered T cells expressing TCRs specific to naturally processed MCPyV T epitopes led to tumor regression in mouse MCC xenografts (Gavvovidis et al., 2018). In one human case study, a similar infusion procedure using MCPyV T antigen-specific T cells that were expanded *ex vivo* resulted in HLA upregulation, T cell recruitment, and responses in most metastases (Chapuis et al., 2014). A recent study developed a means of improving autologous CD8 T cell therapeutic vaccines for MCC by exposing those T cells to cytokine-conditioned dendritic cells presenting LTT peptides on both MHC-I and II surface receptors (Gerer et al., 2017). MCPyV-positive tumor microenvironments can also be engaged by engineered CD4 T cells that recognize the LXCXE epitope of LTT (Longino et al., 2019).

## Immune Responses to MCPyV

Given that the risk factors for developing MCC, including advanced age, UV exposure, and compromised adaptive immunity, can each alter the immune environment in the skin, it is possible that unmitigated proliferation of MCPyV encourages oncogenesis (Stang et al., 2018). The possibility that uncontrolled MCPyV activity leads to MCC cases agrees with the ability of Chang and Moore to predict the viral etiology of MCC due to its greater incidence in immune compromised persons (Feng et al., 2008). The transition from low-level MCPyV persistence to rampant proliferation could promote carcinogenesis by increasing the frequency of integration events or entry into the original host cell of MCC. Because nascently transformed cells are also favored by loss of normal immunity, it would be easy to conflate these two factors. Interestingly though, there is increasing evidence that alterations to systemic and skin immunity can exacerbate MCPyV infections.

A study in Japan found that MCPyV DNA prevalence and viral load on sun-exposed skin increased sharply in individuals over the age of 40 and remained high for the oldest groups (Hashida et al., 2016a). Also, HIV-positive men more frequently have detectable MCPyV DNA on their skin and in their sera, and those with poorly-controlled HIV infection have higher MCPyV antibody titers and DNA loads than those with better-controlled infections (Wieland et al., 2011; Fukumoto et al., 2013; Vahabpour et al., 2017). In kidney transplant recipients, MCPyV DNA was more readily detected in the urine of those with BKPyV-DNAemia and with histologically verified polyomavirus-associated nephropathy (Signorini et al., 2014; Wang et al., 2019). MCPyV DNA detection rates and loads were significantly lower in patients with psoriasis compared to the skin of healthy volunteers suggesting a potential inverse relationship between chronic inflammation in the skin and MCPyV proliferation (Hashida et al., 2019). MCPyV-positive MCC patients, however, have higher MCPyV DNA loads shed from their skin and more frequently produce MCPyV VP1-specific circulating antibodies and at higher titers (Pastrana et al., 2009, 2012; Faust et al., 2011; Touze et al., 2011; Hashida et al., 2016b). More recently, it was found that higher MCPyV DNA load correlates with worse survival outcomes in MCC patients (Von Der Grun et al., 2019).

**Figure 2:**
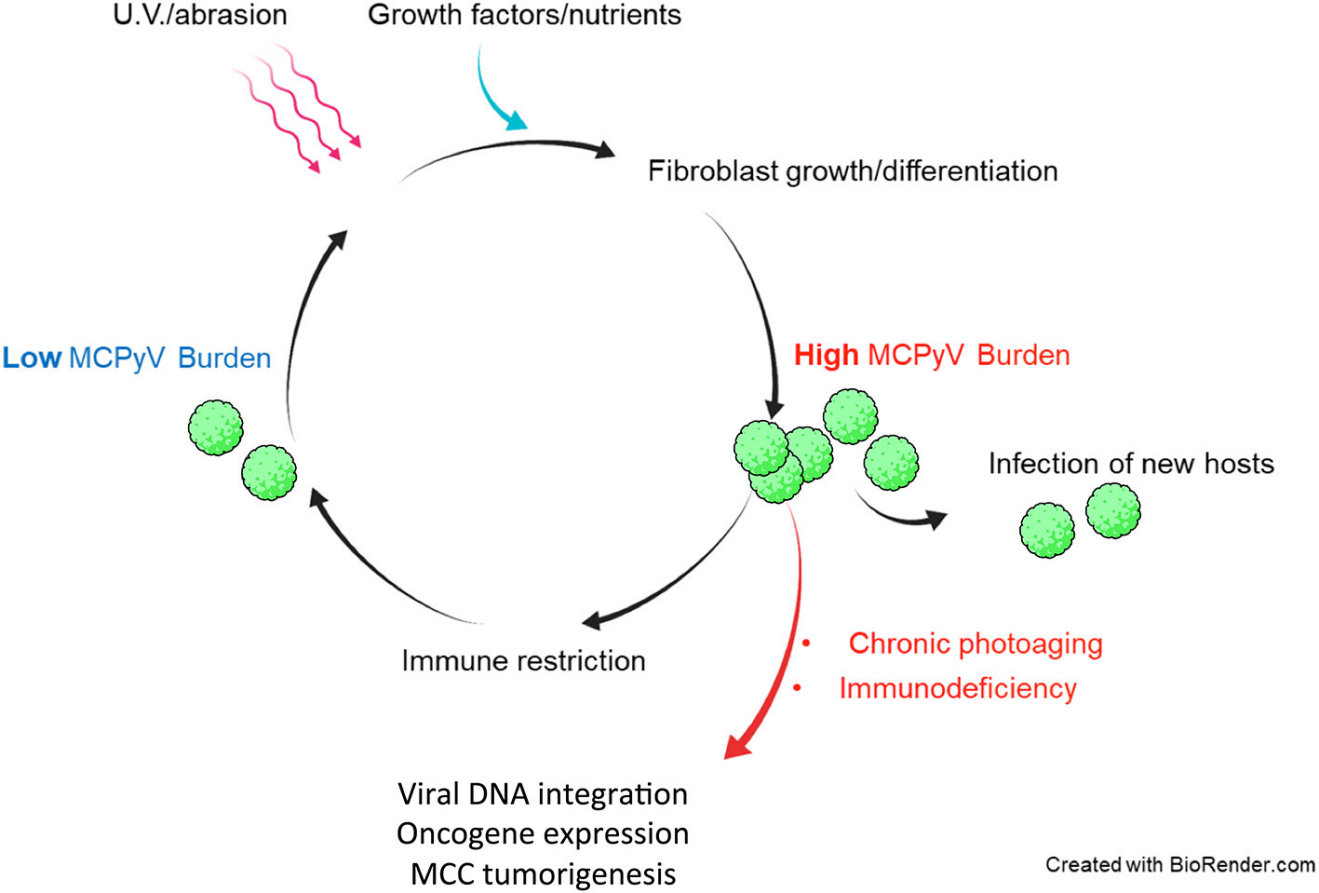
Hypothetical model of *in vivo* MCPyV persistence and dysbiosis leading to MCPyV-positive MCC. Healthy individuals support both low and high MCPyV loads, depending on external factors. U.V. irradiation or abrasion of the skin could cause infected dermal fibroblasts to upregulate MCPyV gene expression and replication. Damaged and repairing skin could be a path by which MCPyV escapes the dermis to infect new hosts. Healthy immune responses may reduce the MCPyV burden asymptomatically. In immunocompromised patients and those with years of chronic U.V. damage, the microenvironment of the skin may be altered in a way that induces MCPyV entry into the original cell of MCC and integration into the host chromatin to drive MCC tumorigenesis.

These findings suggest that sudden loss of normal immune functions could swing the balance between host and virus to favor uncontrolled MCPyV proliferation (Figure 2). From the perspective of the virus, however, MCPyV must have evolved mechanisms of its own to evade host immunity since it can persist and remain highly prevalent in the general population asymptomatically (Tolstov et al., 2009; Schowalter et al., 2010; Foulongne et al., 2012). The mechanisms by which MCPyV evades immune detection and/or destruction are not known.

Some examples of MCPyV gene products or transfected MCPyV genomes interacting with cellular immunity have been documented. For instance, LT expression reduces TLR-9 expression via downregulation of C/EBP transcription factors in epithelial and MCC cell lines (Shahzad et al., 2013). This observation agrees with *in vivo* data revealing that TLR-9 expression was significantly lower in MCPyV-positive MCC tumors compared to MCPyV-negative tumors (Jouhi et al., 2015). MCPyV sT expression was shown to inhibit NF-κB inflammatory signaling through its interaction with PP4C and the adapter NEMO (Griffiths et al., 2013; Abdul-Sada et al., 2017). In addition, it has been shown that a MCPyV-miR-M1 mimic specifically targeted SP100 for degradation (Akhbari et al., 2018). This downregulation of SP100 reduced the secretion of CXCL8 in MCC cells treated with TNF-α (Akhbari et al., 2018). Notably, depletion of the nuclear protein SP100 also enhances MCPyV replication in H1299 cells (Neumann et al., 2016).

There are also examples of cells eliciting an immune response to the expression of MCPyV genes and genomes. As mentioned previously, MCPyV LT and sT expression in BJ-hTERT cells led to upregulation of cellular growth and expression of genes that increase motility (Richards et al., 2015). Accompanying this phenotype was the dramatic induction of many inflammatory response genes including interferon-stimulated genes (ISGs) like OAS1 and ISG20; cytokines like IL-1β and IL-6; and chemokines like CXCL1 and CXCL6 (Richards et al., 2015). Whether MCPyV T antigens induce such a response in the context of infection remains undetermined.

Despite the recent progress made in revealing the MCPyV-host interface, the approaches used previously involved transfection or transduction of MCPyV genes into established cancer cell lines. In order to understand how these mechanisms may contribute to MCPyV persistence, our group examined the innate immune response to MCPyV in the context of infection in human dermal fibroblasts, which plausibly model MCPyV infection of human skin (Krump et al., 2021). After establishing the MCPyV gene expression and replication kinetics in infected cells, we found that late events in the infectious cycle activated the cGAS-STING and NF-κB pathways and subsequent expression of anti-viral ISGs as well as innate inflammatory cytokines (Krump et al., 2021). CRISPR knockout of elements of these immune regulatory pathways yielded significantly higher levels of MCPyV replication per cell, suggesting that the innate gene induction has the potential to restrict MCPyV replication, even in the absence of cellular immune factors (Krump et al., 2021).

## A Model of MCPyV Dysbiosis Leading to MCC Development

MCPyV infects and sheds from the skin of most people without discernible symptoms (Pastrana et al., 2009; Schowalter et al., 2010). The evolutionary strategies that allow MCPyV to asymptomatically infect a large portion of the population, often for prolonged periods of time, remain a mystery (Chen et al., 2011). The auspicious discovery that human dermal fibroblasts support MCPyV infection enabled our recent characterization of the MCPyV infectious cycle and the consequences it poses for the host cell (Liu et al., 2016b; Krump et al., 2021). By understanding the scenarios in which MCPyV might fail to strike a balance with the host immune system, we may be able to infer the events preceding MCPyV integration and oncogenesis.

While direct observation of the interface between MCPyV and systemic immune responses awaits development of an animal model, it is currently possible to formulate a model of MCPyV infection based on data from several sources: (1) the epidemiology of MCC and MCPyV infections, (2) existing research on fibroblast biology and wounding responses in human skin, and (3) *in vitro* findings that establish the prerequisites for MCPyV replication and gene expression (Liu et al., 2016b; Krump et al., 2021). In a similar manner, by juxtaposing decades of skin pathology research with observed cellular response to MCPyV infection, we may be able to provide new insights regarding dysregulated conditions that might lead to MCPyV-driven MCC (Walder et al., 1971; Hardie et al., 1980; Ahmad et al., 2014; Krump et al., 2021).

If indeed dermal fibroblasts are capable of supporting MCPyV infection *in vivo* as they are *ex vivo* and *in vitro* (Liu et al., 2016b), then a model of the course of MCPyV infection can begin to take shape (Figure 2). For instance, healthy human hosts may support a low basal rate of MCPyV activity in fibroblasts that avoids immune recognition, as early events like viral entry and trafficking *in vitro* failed to activate ISGs (Krump et al., 2021). In the event that host skin is abraded or irradiated with UV light, damaged keratinocytes release growth factors and WNT agonists to induce MMP expression and expansion of fibroblasts (Gill and Parks, 2008; Whyte et al., 2012). These tissue changes could stimulate MCPyV early gene expression and DNA synthesis (Liu et al., 2016b). In this scenario, MCPyV PAMPS and/or DAMPs present during later stages of infection upregulate ISGs and inflammatory cytokines in manner that restricts viral replication (Krump et al., 2021). Serological evidence suggests that antibody-related adaptive responses to elevated MCPyV loads could also be a significant restriction factor (Faust et al., 2011). Dermal fibroblasts proliferating and navigating to the wounded tissue or shedding layers of sunburned skin could facilitate MCPyV transmission to new hosts (Figure 2). The antiviral state conferred by ISG induction, innate cytokine signaling, and likely recruited immune cells at the wound site would restrict and clear cells with high levels of MCPyV infection. A return to persistent, low-level MCPyV infection could be achieved by those infected resident skin cells that avoid immune detection (Figure 2).

**Figure 3:**
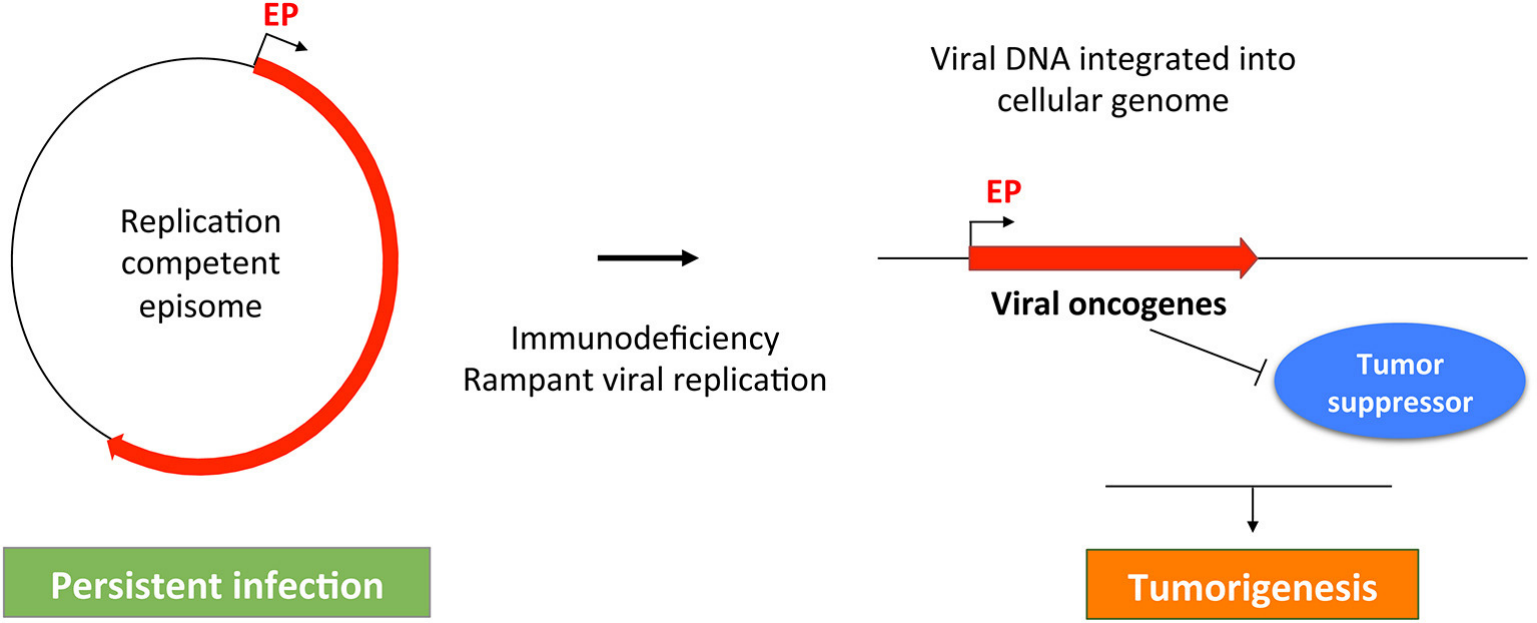
A common theme in MCPyV- and HPV-induced tumorigenesis. MCPyV and HPV both replicate as episomes in persistently infected cell. Failure of host immune system may cause uncontrolled viral replication, which can stimulate viral DNA integration into the host cellular genome. The integrated viral genome expresses viral oncogenes that can inhibit tumor suppressors to induce malignant transformation. Red line indicates the early region of the oncogenic viruses. EP, Early promoter.

Fluctuations in MCPyV load in dynamic equilibrium with insults to the skin and immune responses could be evolutionarily advantageous to MCPyV as it would only risk detection when the opportunity to infect new hosts arises (Figure 2). Should MCPyV activity correspond to skin damage and repair, then chronic wounding, inflammation, or altered skin architecture could represent the early events that promote MCPyV DNA amplification and integration into host DNA that lead to MCPyV-associated MCC (Figures 2, 3). This possibility is supported by epidemiological studies showing a strong correlation between immunosuppression, elevated MCPyV genome loads, and increased risk for MCC (Heath et al., 2008; Wieland et al., 2011). These studies suggest that faulty immune control of MCPyV infection could disturb the balance of MCPyV-host interaction to cause unbridled viral replication, which can increase the chance of incidental integration of viral DNA and MCC oncogenesis (Figures 2, 3). These observations would also explain why certain populations have a higher risk of developing MCC.

The molecular events linking MCPyV infection and MCC development are reminiscent of those underlying the malignancies driven by human papillomaviruses (HPVs). In non-malignant human cells, HPVs, like MCPyV, normally replicate and maintain their genomes as episomes (Longworth and Laimins, 2004; You et al., 2004; You, 2010; Wang et al., 2013) (Figure 3). During the course of persistent infection, a compromised immune system and/or other pathologic conditions could cause rampant viral replication to promote integration of viral genomes into the host DNA. An additional parallel between MCPyV and HPV oncogenic mechanisms is that the integrated viral genomes typically lose the ability to replicate, but the non-replicating viral genomes retain the capacity to express viral oncogenes, such as LTT/sT (encoded by MCPyV) and E6/E7 (encoded by HPV). These viral oncoproteins stimulate cellular proliferation and malignant transformation by inhibiting host tumor suppressor such as RB and p53 (Gaglia and Munger, 2018) (Figure 3). The uncontrolled cellular proliferation could ultimately inflict oncogenesis by allowing virally induced precancerous lesions to persist and expand (Figure 3). In addition, viral oncogenes encoded by the integrated MCPyV and HPV genomes also share the ability to induce genomic instability (Li et al., 2013; Gaglia and Munger, 2018), which can introduce more DNA breaks in the host genome to stimulate viral genome integration. Hyperproliferation and DNA damage induced by viral oncogenes and/or U.V. irradiation may allow the emerging tumor cells to accumulate additional genetic mutations needed to develop into invasive tumors (Figure 3).

## Future Perspective

Over 90% of MCC patients are not immune-compromised by clinical definitions, yet almost all are over the age of 50 and have low melanin content in their skin (Heath et al., 2008). Therefore, unraveling the impact of UV-radiation and aging to the skin could reveal key aspects of early events in MCPyV-associated MCC. Further elucidation of tactics employed by MCPyV to manipulate the host immune system for promoting its own propagation and driving cellular transformation will likely offer new clues for understanding the mechanism driving MCPyV tumorigenesis.

The bourgeoning of cancer therapies aimed at activating and directing immune responses to malignancies give much cause for hope for the treatment of MCC and other viral cancers. Still, gaps in our understanding of the biology driving MCPyV-related oncogenesis and MCC immune escape could widen the breadth of patients that respond to treatments and the durability of those responses. Addressing some of our gaps in knowledge will require technological advances like improved detection of low-copy number viral DNA genomes in tissue isolates or accurate animal models of MCPyV infection. Until that time, we can make strides in our investigation using the wealth of knowledge on human skin and cancer biology, and by expanding the model of *in vitro* MCPyV infection. Applying this knowledge to re-establish asymptomatic equilibrium between host and MCPyV in at-risk individuals has the potential to prevent MCC cases from occurring in the future.

## Author Contributions

NK and JY: conceptualization and writing—review and editing. NK: writing—original draft preparation and visualization. JY: supervision, project administration, and funding acquisition. Both authors have read and agreed to the published version of the manuscript.

## Funding

This work was supported by the National Institutes of Health (NIH) Grants R01CA187718, R21AR074073, R21AI149761, the NCI Cancer Center Support Grant (NCI P30 CA016520), and the Penn CFAR pilot award (P30 AI 045008).

## Conflict of Interest

The authors declare that the research was conducted in the absence of any commercial or financial relationships that could be construed as a potential conflict of interest.

## Publisher's Note

All claims expressed in this article are solely those of the authors and do not necessarily represent those of their affiliated organizations, or those of the publisher, the editors and the reviewers. Any product that may be evaluated in this article, or claim that may be made by its manufacturer, is not guaranteed or endorsed by the publisher.
